# The impact of cellular environment on *in vitro* drug screening

**DOI:** 10.2144/fsoa-2023-0027

**Published:** 2023-09-07

**Authors:** Aiman Moldasheva, Laura Bakyt, Denis Bulanin, Mohamad Aljofan

**Affiliations:** 1 Department of Biomedical Science, Nazarbayev University School of Medicine, Astana, 010000, Kazakhstan; 2 Drug Discovery & Development Laboratory, Centre of Life Sciences, National Laboratory Astana, Nazarbayev University, Astana, 010000, Kazakhstan

**Keywords:** cell culture, drug screening, microenvironment, oxidative tension, pH, ROS

## Abstract

There are various reasons for drug failure in the developmental stage including toxicity, adverse effects and inefficacy. This is likely due to the differences in drug behavior between a simple and controlled cell culture system to that of a more complex whole organism environment. While the use of human phenotypical cells relevant to the condition may provide more accurate screening results, they are susceptible to producing false positives as cells are continuously influenced by constant chemical and physical interaction with the surrounding microenvironment. Therefore, several microenvironmental and pharmacomechanical aspects must be factored in during tissue culture drug screening.

## Plain language summary

Drug discovery is a lengthy process that goes through several preclinical and clinical testing stages. One of the earliest stages in preclinical testing involves testing new drug using isolated cells. This system is an important tool in research and a commonly used technique in drug testing. However, a number of subtle, but important issues appear to affect its results. Therefore, in this review, we attempt to address some of the important issues that could lead to false positive or negative hits.

There are many reasons why successful hits fall short of reaching the clinical development phase. While toxicity is considered to be the major reason for halting drug development, many studies cited inadequate drug efficacy as one of the most commonly reported reason for drug failure [1]. Recent estimates showed that more than half of drugs fail in clinical trials due to lack of efficacy [2]. A report that analyzed a five-year period (2005–2010) for AstraZeneca portfolio performance of drug failure, suggested that more than 70% of developmental drugs fail in stage II studies and less than 10% in preclinical stage [3]. One of the significant contributors thought to have influenced the rate of failure in drug discovery is the initial drug screening methodologies including cell culture. There is mounting evidence suggesting that cells cultured in the non-physiological 2D conditions are not representative of cells residing in the complex microenvironment of a tissue [4]. This issue is partly addressed by using more complex systems resembling *in vivo* conditions such as 3D cell cultures, microfluidic devices and models using 3D bioprinting [5]. Whilenewer screening systems appear to be more representative of physiological conditions, their usage is also not without disadvantages. Some of the drawbacks are high cost, labor intensity, absence of standardized protocols, low reproducibility and imaging difficulties [6,7]. Therefore, 2D cell culture remains to be the most commonly used approach worldwide for *in vitro* screening due to its time- and cost–effectiveness [8]. Therefore, understanding factors that can affect the reproducibility of 2D cell culture drug screening is essential to avoid a high attrition rate later in drug development. There are several environmental factors that need to be controlled or observed in the *in vitro* models, which can influence cellular response and behavior. These factors include glucose concentration in the extracellular environment, oxygen tension, the extent of oxidative stress, pH level of the media and others. The ability to control these environmental parameters becomes especially significant for anti-cancer drug screening. Firstly, anti-cancer drugs have a very low rate of clinical development success compared with other drug classes [9], which is believed to be mostly due to inefficient pre-clinical *in vitro* screening. Secondly, the tumor microenvironment plays a tremendous role on cell growth, proliferation and overall tumor fate [10]. Therefore, it is necessary to identify and control the cellular environmental factors that could impact the overall cellular response to make initial drug screening results more reliable. In this review, we discuss some of the important *in vitro* environmental factors that would impact drug screening with a particular focus on *in vitro* anticancer drug screening.

## Effect of glucose level on cancer cells

**Figure 1.**
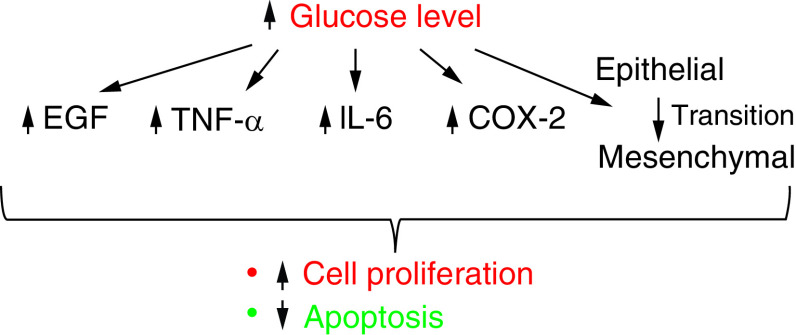
Increased glucose level is associated with increased expression of EGF, TNF-α, IL-6, COX-2 and transition of epithelial cells to mesencymal cells – all of which can contribute to increased cellular proliferation and decreased apoptosis.

Hyperglycemia has been implicated as a factor for cancer development and that people with diabetes have an increased risk of several common cancers [11]. More than half a century ago, Warburg *et al.*, suggested an association between glucose level and cancer development through what is now known as the “Warburg effect” where cancer cells reprogram their metabolism to promote growth, survival and proliferation [12]. Hou *et al.*, claimed that hyperglycemia might serve as a means of nutrients supply that would increase proliferation of malignant tumor cells as they showed that increasing glucose levels of breast cancer cells from 5 mM to 25 mM resulted in a significant increase in cellular proliferation [13]. This is not an uncommon finding with a number of studies have highlighted the importance of cellular glucose level for drug screening, particularly for the activity of anticancer candidates. For instance, in an attempt to determine the anticancer mechanism of metformin, Liu *et al.*, claimed that antiproliferative effects of metformin in cancer cells are highly dependent on the glucose concentration in the extracellular environment [14]. There are numerous factors that could explain the effect of high glucose on cellular environment including the increase in the expression of epidermal growth factor (EGFR), which thought to increase the cancer cell proliferation [15]. Also, chronic hyperglycemia stimulate the production of several cytokines including tumor necrosis factor-α (TNF-α), interleukin-6 (IL-6), as well as cyclooxygenase-2 (COX-2) [16,17], all of which were shown to promote tumor cell proliferation, inhibit cellular apoptosis regulate cell cycle, and induce the expression of oncogene [18] (Figure 1). Furthermore, prolonged elevation of glucose level was reported to promote the transition of epithelial to mesenchymal state, which increases cancer progression, cellular invasion and inhibit cellular apoptosis [19].

To the contrary, a reduction in the cellular glucose level was found to significantly affect the anticancer activity of investigational drugs. A recently published study by Ma *et al.*, who investigated anticancer activity on ovarian cancer cells, claimed that low glucose environment enhances ovarian cancer cell cytotoxicity [20]. Indeed, low glucose/starvation can lead to cellular apoptosis via many different pathways including autophagy, which is thought to partly occur through the activation of AMP-activated protein kinase (AMPK)/mechanistic target of rapamycin (mTOR) energy sensing pathway [21]. It was hypothesized that glucose or glucose derived molecules maybe involved in anti autophagic signaling, thus glucose deprivation would likely result in cellular autophagy [22].

## Effect of oxidative stress

Reactive oxygen species (ROS) or radicals are consistently produced as part of metabolic processes during biochemical reactions in living cells [23]. The production and increase in ROS concentration leads to the formation of oxidative stress, which also occurs due to insufficient protection of the body by antioxidants. ROS can be either endogenously, or exogenously produced and results in oxidative stress that can either occur in an acute or chronic form [23]. Ahmed *et al.*, suggested that oxidative stress in its short-term occurrence might lead to ischemia-reperfusion syndrome, an acute inflammation and hyperoxia that is not commonly associated with tumor development, but chronic oxidative stress is strongly associated with inflammation and cancer occurrence [24]. During inflammation, cytokines stimulate the production of superoxide (O_2_‘), nitric oxide (NO’), and hydrogen peroxide (H_2_O_2_) that serve as a form of defense against viral, bacterial or parasitic infections.

However, the oxidation may lead to DNA damage and, thus, tumor development. Chronic oxidative stress affects the growth of tumorous cells, DNA damage, chemotherapy resistance resulting in metastatic progression and invasion [25]. Continuous oxidative stress contributes to the development of chemotherapeutic resistance and protein damage leading to cancer invasion. Yet chronic oxidative stress, on its own considered insufficient to cause cell death, as cancer cells happen to have low level of responsiveness to oxidative stress due to increased antioxidant resistance by cellular membrane and DNA [24]. The effect of oxidative stress caused by ROS is seen in every stage of carcinogenesis [24].

In addition, living cell have a capability to maintain all damage caused by ROS-induced stress, through nuclear excision repair mechanism, and repair of specific lesions involving various enzymes [24]. Cell culture is the most utilized model of study for drug screening including the investigation of drug effect, crude toxicity, and molecular mechanisms. While the usual O_2_ level in *in vitro* cell culture is approximately 21%, an increase in ROS in cell culture may influence cellular physiology. An increase in the O_2_ level of more than 21%, will likely affect the *in vitro* results [26]. Also, photochemical oxidation of flavonoids or polyphenolic compounds increase H_2_O_2_ production in cell culture media, leading to an increase in ROS production [26]. The oxidative stress in cell culture may appear due to either high level of ROS generation, or due impairment of cellular defense against particular antioxidants [26]. Halliwell *et al.*, claimed that the results of elevated ROS formation increase cell growth, halt cellular proliferation, senescence, cell death, or adaptation, and that adaptive and proliferative features of the cell might be changed [27].

Additionally, the instability of O_2_ levels in cell culture, changes from high to low during cell growth and proliferation, changes the cellular status from hypoxia to hyperoxia, which may result in hypoxia-reperfusion damage [27]. The accumulative ability of ROS in living cells is related to occurrence of stress in the endoplasmic reticulum (ER), which has a dynamic structure that is involved in the protein synthesis, lipid metabolism, calcium storage and monitoring of appropriate protein folding. Thus, an increase in the concentration of ROS leads to the disruption of the functionality of the ER [28], and depending on the duration, a stressed ER may stimulate cellular apoptosis. Another player in ROS generation is H_2_O_2_, which is transformed from superoxide. Several studies showed that oxidative stress caused by addition of H_2_O_2_ to different cell cultures (HEK293, HeLa and CHO cells) could cause adverse impact on cellular proliferation and viability [27]. Ha *et al.*, demonstrated that H_2_O_2_ involvement in rCHO cell cultures caused oxidative stress through increase in ROS level and stress condition of ER, which reduced cell growth and viability [28].

Furthermore, there appears to be a significant link between oxidative stress and neurodegenerative diseases due to the unstable cellular response to oxidative stress, which might be a reason of hyperphosphorylation of cytoskeletal proteins related to several neurodegenerative disorders. Egaña *et al.*, argues that phosphorylation state of tau protein both in its increase or decrease, takes a specific place in response to oxidative stress. For example, dephosphorylation of tau protein was seen in rat primary neuronal cultures that was caused by oxidative stress after addition of H_2_O_2_ [29]. Thus, there is a significant impact of ROS on cell culture that needs to be control or taking into account during *in vitro* drug screening.

## Effect of oxygen tension

Oxygen tension is one of the important components involved in regulation of different developmental processes. Tsiapalis *et al.*, claimed that tissues undergo through broad spectrum of oxygen tension *in vivo*, which depends on its place of occurrence and difference in capillary supply [30]. Diffusion range between 100 and 200 μm is considered to be optimal for cell oxygenation. Over the period of prenatal development, hypoxia acts as a significant stimulus that is necessary for adequate patterning and functioning of various body organs [31]. Oxygen tension plays significant part in a number of diseases and the deficiency in capillary development in adipogenesis, which leads to hypoxia and further inflammatory processes [32]. A hypoxic environment could affect a number of different diseases from cancer to dementia, and diabetes [31].

For example, the impact of oxygen tension is extensively studied in cancer biology, and it is suggested that cancer cells group into spheroids that will subsequently influence the vascular, lymphatic systems, and different surrounding cells [33]. This mass later decreases the level of important supplies for survival. Subsequently, a reduced supply of oxygen, results in chronic or transient hypoxia, which plays a major role in differentiation of physiology and features of tumor, such as drug resistance by hypoxia-inducible factor (HIF) [33]. Wei *et al.*, claim that hypoxia could significantly affect tumor microenvironment that is involved in cancer growth and its resistance to various type of treatment [34]. More than a half of cancer incidents demonstrate hypoxia presence in its tissues. It is stated that cancerous tissues tend to adapt to hypoxia environment to keep alive, promote its progression and metastasis. In consequence those factors give possibility to be resistant to anticancer treatment [34]. The hypoxic environment around cancer cells may influence the efficacy variety of anticancer treatment, with several antitumor drugs demonstrated significant increase of their effectiveness in hypoxic environment. Therefore, oxidative tension of tumor microenvironment is an important factor that needs to be further studied to determine its impact on cancer tissue and for anticancer drug screening [33]. However, understanding the exact mechanism of tumor tissues behavior and oxidative tension *in vivo* is complex due to different physicochemical factors and chemoattractant.

Low levels of oxygen tension during culturing of non-immortalized primary cells or stem cells were reported to affect the replicative feature of the cells, and thus, it is suggested to culture primary cells at low O_2_ levels [26]. Also, an increase of O_2_ concentration might affect the release of free radicals and their reduction can result with hypoxia. Subsequently, both oxidative stress and hypoxia lead to the same outcome, because of the mutual mediator, ROS, even though they differ in its oxidative tension features [26]. The production of ROS in the process of hypoxia or hyperoxia has an impact on epigenetic changes. In the condition of decreased oxygen tension, the specific elements of hypoxia intensify the mRNA protein levels of a number of histone demethylases. Whereas increased oxygen tension in immortalized cells culture could demonstrate the raise in inflammatory response [26]. Lennon *et al.*, reported the impact of decreased oxygen tension on osteoblasts, pericytes and periosteal cells, which have the ability to differentiate into osteoblasts [35]. They claimed that a reduced oxygen tension condition supports the increase in proliferation and the alkaline phosphatase activity of rat marrow-derived mesenchymal stem cells (rMSCc) [35].

In another study by Genbacev *et al.*, who investigated oxygen tension in a culture of cytotrophoblasts, which are known as special type of placental cells that have the ability to proliferate at initial stages of gestation period, and further differentiate to cancer-like cells that create blood stream for the placenta [36]. The authors found out that cells terminate their proliferation and differentiate normally in a condition of culturing at 20% of oxygen tension. Thus, it mimics the milieu of uterine arterioles. Therefore, oxygen tension was shown to define if cytotrophoblasts can normally proliferate and regulate placental growth [36]. D'Ippolito *et al.*, investigated the role of oxygen tension in regulating the balance between self-renewal and differentiation of human marrow stroma-derived cells [37]. They demonstrated that low level of oxygen tension contributes to cartilage formation. While such experiments were previously reported using various cell models, this study utilized a new cell model, MIAMI (marrow-isolated adult multilineage inducible cells) [37], further supporting the significant impact of oxygen tension on cell culture environment.

## Effect of pH level

Cellular pH is an important physiological parameter, which is tightly regulated, and a change in the cellular pH affects nearly all functional aspects of cellular physiology. It was demonstrated that majority of cells have cytoplasmic pH of approximately 7.2 and this pH is constantly maintained [38]. However, organelles within the cells such as mitochondria, lysosomes, endosomes and Golgi apparatus have pH levels different from the cytoplasm and regulate it independently. In a lot of cases this difference in the pH is important for the functional activity of these organelles. Mitochondria, for example, has more alkaline pH (7.5) compared with cytoplasm (7.2) and this pH gradient across mitochondrial membrane is required for its main function – ATP synthesis [39].

Despite the fact that stability of intracellular pH is constantly challenged by cellular metabolism itself and leakage of acidifying factors from organelles, there are working mechanisms in place such as cellular buffering systems and transporters [39]. Intracellular weak acids and bases due to their ability to bind to protons and hydroxyl ions respectively work as a buffering system to protect the cells from sharp changes in the pH. Other important players in maintaining pH homeostasis are membrane transporters such as Na^+^-H^+^ exchangers, HCO_3_^-^ – dependent transporters, H^+^-ATPases, organic anion transporters and others [40].

A lot of cellular functions are affected by change in the cytoplasmic pH including but not limited to: cellular metabolism, cytoskeleton activity, activity of intracellular enzymes, muscle contraction, cell-to-cell coupling and cell growth and proliferation. This becomes very important during tumor development because cancer cells undergo heavy changes in cellular metabolism accompanied by change in the intracellular and extracellular pH [41].

**Figure 2.**
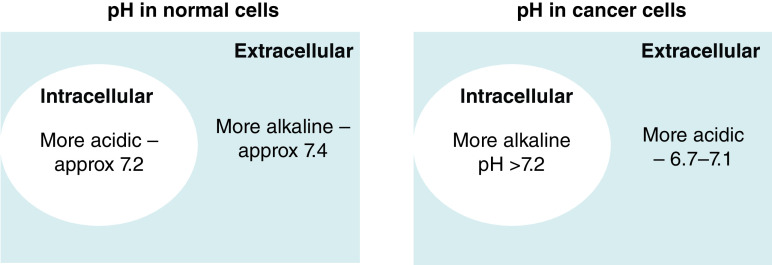
pH reversal in cancer cells compared with normal cells. In normal cells intracellular environment is usually more acidic compared with the extracellular, whereas cancer cells have more acidic extracellular environment compared with intracellular.

It has been demonstrated that cancer cells have more acidic pH in the extracellular environment compared with intracellular, whereas the opposite is true for the normal cells [42] (Figure 2). This deregulation of pH homeostasis in cancer cells happens mostly as a result of switch from oxidative phosphorylation to aerobic glycolysis to produce the energy [41]. Normal cells produce energy in the form of ATP through the citric acid cycle and oxidative phosphorylation, whereas cancer cells use less effective pathway of aerobic glycolysis even in the presence of oxygen [43]. As a result of this cellular metabolic changes, more acids are produced and secreted into extracellular space, resulting in acidification of extracellular environment in the cancer cells [43]. Acidification of extracellular environment confines survival advantage to cancer cells as normal cells cannot grow and differentiate in such conditions [44]. Moreover, extracellular acidosis of cancer cells is believed to play a role in resistance to anticancer drugs [45]. Alkalization of intracellular pH of cancer cells, on the other hand, is also advantageous in terms of their cellular viability. Persi *et al.*, have demonstrated that lowering of the intracellular pH (making it more acidic) can be detrimental for survival of cancer cells and can become potential target mechanisms for anticancer drugs [46].

Knowing that reversal of pH gradient is an important hallmark of cancer cells, which not only gives the survival advantage but also can confer resistance to anticancer drugs, it is imperative to maximally reproduce this gradient during the *in vitro* studies. Standard *in vitro* conditions, however, usually utilize physiological pH levels of healthy cells. This can lead to potential false positive results, as an important condition, which can lead to anticancer drug resistance, such as acidic extracellular pH is not reproduced. Moreover, levels of intracellular and extracellular pH need to be constantly monitored as cancer cells have high metabolic rate.

## Conclusion

Despite the emergence of newer *in vitro* technologies, 2D cell culture is still routinely used worldwide for drug screening purposes. Even though it has its disadvantages, it still allows for cheap, fast and robust initial screening. One of the main drawbacks is difficulty to reproduce dynamic physiological environment typical for the cells in the living organism. One possible attempt to solve this problem is to control environmental factors that can affect cellular behavior. This manuscript concentrated on the environmental factors that can affect anti-cancer drug screening because for cancer cells tumor microenvironment is especially important as it can determine tumor fate and response to the drugs. Environmental factors such as glucose concentration, extent of oxidative stress, oxygen tension and level of pH and their potential effect on the cancer cells behavior were reviewed in detail. In conclusion, the use of cell cultures for *in vitro* drug screening must be controlled as much as possible and potential influence of environmental factors must be accounted for during interpretation of results to avoid late drug attrition.

## Future perspective

The advancement in cell science and tissue engineering will enhance cell culture in several ways including, controlling of cellular microenvironment that will improve their resemblance to that of a whole organism. The establishment of organoid systems, *ex vivo* cultures and leveraging of artificial intelligence in cell culture are set to transform the entire *in vitro* system. This will lower the operational cost and shorten the discovery time.

**Executive summary**

- A large number of drugs fail in the preclinical testing stage.
- Glucose concentration, oxygen tension, reactive oxygen species (ROS), and pH are important factors that can affect drug behavior *in vitro*.
- 3D cell culture might provide better resemblance to *in vivo* system than 2D.

**Effect of glucose level on cancer cells**

- *In vitro* glucose level affects cellular growth.
- Chronic hyperglycemia induces the production of cytokines.
- Hypoglycemia induces apoptosis.

**Effect of oxidative stress**

- ROS production may lead to inflammation.
- Oxidative stress leads to DNA damage and chemotherapeutic resistance.
- Increase in oxygen levels may affect drug screening results.

**Effect of oxygen tension**

- Low oxygen levels may inducedrug resistance by hypoxia-inducible factor.
- Cancer cells adapt to hypoxic environments.
- It is vital to control oxidative tension of tumor microenvironment.

**Effect of pH level**

- A change in pH level can affect cellular physiology.
- Cancer cells have a more acidic extracellular environment compared with intracellular.
- Acidic extracellular environment affects anticancer drug activity.
